# Mechanical and Thermal Performance of Macro-Encapsulated Phase Change Materials for Pavement Application

**DOI:** 10.3390/ma11081398

**Published:** 2018-08-10

**Authors:** Xiangming Zhou, Gediminas Kastiukas, Claudio Lantieri, Piergiorgio Tataranni, Rosolino Vaiana, Cesare Sangiorgi

**Affiliations:** 1 Department of Civil and Environmental Engineering, Brunel University London, London UB8 3PH, UK; xiangming.zhou@brunel.ac.uk; 2 DICAM Department, University of Bologna, 40136 Bologna, Italy; claudio.lantieri2@unibo.it (C.L.); piergiorg.tataranni2@unibo.it (P.T.); cesare.sangiorgi4@unibo.it (C.S.); 3 DINCI Department of Civil Engineering, University of Calabria, Arcavàcata di Rende, 87036 Cosenza, Italy; rosalino.vaiana@unical.it

**Keywords:** lightweight aggregates, phase change materials, macro-encapsulation, thermal performance, crushing test, road application

## Abstract

Macro-encapsulated phase change material (PCM) lightweight aggregates (ME-LWA) were produced and evaluated for their mechanical and thermal properties in road engineering applications. The ME-LWAs were first characterised in terms of their physical and geometrical properties. Then, the ME-LWAs were investigated in detail by applying the European Standards of testing for the Bulk Crushing Test and the Polished Stone Value (PSV) coefficient as well as Micro-Deval and laboratory profilometry. In addition, the thermal performance for possible construction of smart pavements with the inclusion of ME-LWAs for anti-ice purposes was determined. The crushing resistance of the ME-LWAs was improved, while their resistance to polishing was reduced. Thermal analysis of the encapsulated PCM determined it to possess excellent thermal stability and a heat storage capacity of 30.43 J/g. Based on the research findings, the inclusion of ME-LWAs in surface pavement layers could be considered a viable solution for the control of surface temperatures in cold climates. Road safety and maintenance could benefit in terms of reduced ice periods and reduced treatments with salts and other anti-ice solutions.

## 1. Introduction

Road surfaces, pavements and buildings all contribute to keeping urbanised environments three to four degrees warmer than surrounding non-urbanised areas, an issue that leads to the creation of so-called urban heat islands (UHIs). Dense dark surfaces such as bitumen on roads and building materials are the primary contributors since they accumulate and store heat during the day and then release it at night. A common strategy to reduce the UHI effect is to provide more shade with increased tree canopies. However, such passive methods have limitations due to the low availability of space in urban areas. One of the most effective active methods to reduce the heat accumulation is to incorporate a phase change material (PCM) as an active additive. PCMs have high latent heat storage densities and can, therefore, absorb thermal energy when transforming from solid to liquid or release it when turning back to a solid [1], thus maintaining constant temperature performance. PCMs have good insulation heat storage performance and have been used widely in the construction, renovation and energy-saving industries, especially for buildings [2,3]. However, their application in the road transport sector is still at the exploratory research stage and may introduce benefits from a road safety point of view. Here moisture control, in particular ice formation on the pavement surface, is addressed.

By means of an experimental analysis, Chen et al. [4] proved the feasibility of using PCMs in asphalt mixes and put forward the basic technical selection criteria used in asphalt mixes with PCMs. Ma et al. [5] conducted field simulation tests of asphalt mixtures with PCMs, concluding that they helped to decrease the highest temperature during the heating process and increase the lowest temperature during the cooling process, which reduced the temperature’s adverse impact on asphalt mixtures. The test results indicated that an asphalt mixture with PCM actively influenced the pavement temperature with a damping effect, and also enhanced the temperature resistance capacity for asphalt layers, as well as improved the asphalt mixture adaptability to the changing environment. 

The incorporation of PCMs into construction materials depends on the material properties and can be primarily achieved in the following three ways: (1) direct incorporation at the time of mixing, which has been successfully implemented in frame walls [6]; (2) immersion of the component in liquid PCM: a technique proven successful in concrete [7] and (3) micro-encapsulation [8] The method of encapsulation is the most advanced and popular because it allows for better dispersion, reduces the external volume changes and reduces the possibility of PCMs leaching into the surrounding material by eliminating the direct interaction between PCM and host material [9]. Micro-encapsulation can be achieved through physical processes such as spray cooling [10], spray drying [11], and the fluidized bed processes [12]. Chemical processes are also used and include in situ polymerization (interfacial polycondensation, suspension polymerization, and emulsion polymerization), complex coacervation, sol-gel method, and solvent extraction/evaporation method [13]. Physical methods of production are limited by their granulated sizes thus chemical methods are frequently employed to produce much smaller encapsulated PCM particles. Furthermore, Hawlader et al. [14] reported a substantial drop in heat storage capacity with the physical methods of production. Although PCM microcapsules have been produced on an industrial scale, the production process is very expensive, thus limiting their use to low volume incorporation applications such as textiles [15].

Alternatively, PCMs can also be encapsulated on a macro scale via a porous host material. In the study by Kastiukas et al. [16], liquid paraffin was forced into porous, lightweight expanded clay aggregates (LWA) using a vacuum impregnation system, similar to that used by Memon et al. [17]. This method proved to be simple to set up and allowed the impregnation to be precisely controlled.

Thus, the purpose of this study was to implement the authors’ existing technological advancements of ME-LWAs for application in road pavements. ME-LWAs are evaluated for their mechanical and thermal suitability for pavements. Nonetheless, it is envisaged that the knowledge resulting from this study could be transferred to applications in concrete, mortar, grout, bituminous mixtures and surface treatments for unbound and hydraulically bound applications in construction works.

## 2. Materials and Methods

### 2.1. Material Testing

To produce the ME-LWAs, LWAs (Argex S.A, Bustos, Portugal) conforming to EN 13055-1 were used. The diameter of ME-LWA was determined using electronic callipers and was recorded as the average of three measurements per specimen, for a total of 10 specimens. The shell thickness was determined under the inspection of a light microscope and was taken as the average of 10 samples. 

The average dry particle density of the ME-LWAs was determined in accordance with EN 1097-3 by sand pycnometry. 100 ME-LWAs were put into a flask and covered with a known amount of fine sand to measure the volume. The particle density was calculated by dividing the mass of the material by the measured volume.

The LWA water absorption was determined in accordance with EN 1097-6.

The thermal conductivity of the raw LWA was measured in accordance to (EN 12664), using a Netzsch heat flow meter (HFM 436 Lambda) (Netzsch Gerätebau GmbH, Selb, Germany) heat flow meter with a hot plate and cold plate set at 35 and 15 °C, respectively.

Differential scanning calorimetry (DSC) analysis was adopted to evaluate phase changing behaviour, i.e., phase change temperature and thermal energy storage. The ME-LWA was crushed to a coarse powder, which was used to conduct the DSC test on a Q2000 (TA Instruments, New Castle, DE, USA). The DSC sample weight was approximately 10 mg. Several samples were tested, implementing two measuring cycles in a temperature range of −20 °C to +80 °C, at a heating/cooling rate of 5 °C/min. Calculations for the stored/released thermal energy in unit weight of the material, also known as specific phase change enthalpy (ΔH_m_/ΔH_c_) were calculated by the DSC software TRIOS v4.3.1.

Other mechanical and physical tests encompassed the bulk crushing resistance, the Polished Stone Value, the Micro-Deval resistance to fragmentation, the laser profilometry and the thermal behaviour under relevant temperature external conditions and chamber controlled temperature drop. The latter was reproduced using existing data from a local climate station installed at the Bologna International Airport.

### 2.2. PCM Impregnation and Coating

The LWAs were sieved to obtain a maximum particle size of 8 mm. This limit was chosen in consideration of a potential increase of 1 mm in particle diameter after coating, determined from previous research [16]. The LWAs were also blow-dried with compressed air to remove surface dust before impregnation. The absorption capacity of the LWA was determined by using the mass change of the PCM-impregnated LWA and the PCM density of 0.77 g/mL and an average LWA intrusion volume of 0.82 mL/g (determined using Mercury Intrusion Porosimetry (MIP)). For comparison, normal immersion of the LWA into PCM was also evaluated. The absorption capacity of normally immersed LWAs was only approximately 10% of that reached using vacuum impregnation.

Technical grade paraffin (Rubitherm GmbH, Berlin, Germany) was chosen as the PCM for the impregnation of LWAs. The thermophysical properties of the PCM are reported in Table 1.

To prevent the leakage of PCM from the pores of the LWA, the impregnated aggregates were coated using a commercial-grade polyester resin Palatal P4-01 (Aliancys AG, Schaffhausen, Switzerland), with a novel method developed in the previous research of [16]. Polyester resin was chosen due to its high surface hardness, stiffness, compressive and tensile strength. Some of the resin properties are reported in Table 2. The mixing ratio of adhesive:harder:catalyser, which would provide the most manageable working time, in this case, 15 min, was determined to be 1:0.02:0.03 by mass. Due to the high viscosity of the resin, granite powder was used as the final step of the coating procedure to prevent agglomeration of the ME-LWAs. The granite powder was also intended to increase the roughness of the surface, helping the ME-LWA interlock with a potential matrix during hardening and provide better aggregate-paste bond strength.

**Figure 1:**
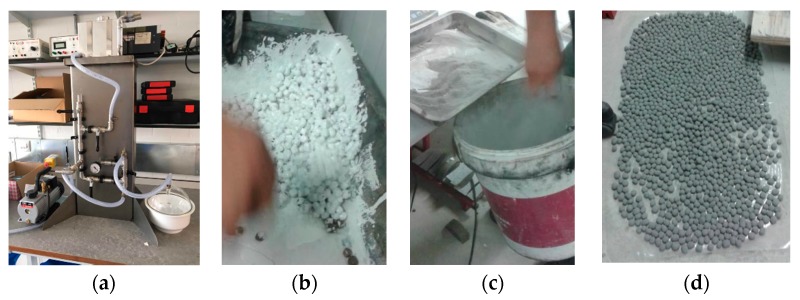
PCM impregnation and coating showing (**a**) the vacuum impregnation system (**b**) resin coating the PCM-LWA (**c**) introduction of granite powder, and (**d**) final ME-LWAs.

PCM was introduced into the pores of the LWA using an in-house vacuum impregnation system (Figure 1). Weighed samples of LWA were placed into vacuum chambers and sealed using vacuum gel. Air entrapped within the pores of the LWA was removed under a vacuum pressure of −860 mbar for 30 min. Liquid paraffin was then allowed to enter the chambers and completely submerge the LWAs. The air was then allowed to enter the chambers to help force the paraffin into the pores. After this, the sample was left to rest for a further 30 min. Upon completion of the impregnation process, the PCM-LWAs were surface-dried using absorbent towels to remove excess paraffin and immediately placed into an environmental chamber maintained at a temperature below the phase change temperature to keep the PCM in a solid state.

All the tests were conducted with ME-LWA samples that had been oven-dried in accordance with EN 1097-5 and subsequently conditioned at 23 ± 5 °C to allow the test specimen to cool to room temperature. Sampling of the ME-LWAs was conducted in accordance with EN 932-1.

## 3. Results and Discussion

### 3.1. Geometric and Physical Characteristics of ME-LWAs

**Figure 2:**
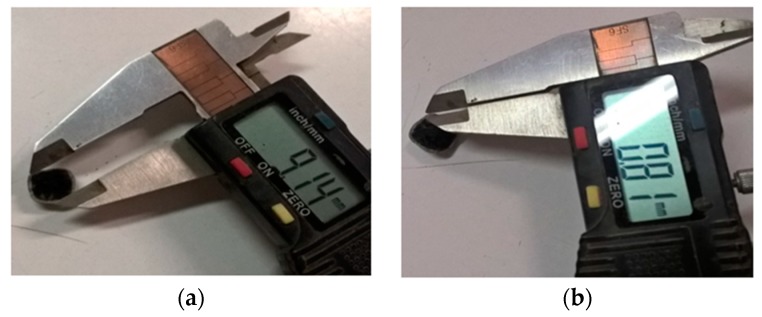
Measurement of (**a**) ME-LWA diameter and (**b**) ME-LWA coating thickness.

**Figure 3:**
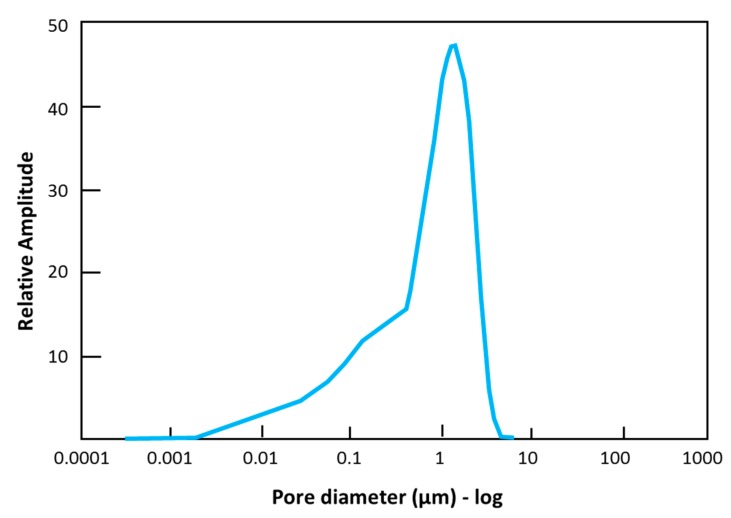
Pore size distribution of the LWA.

Figure 2 shows a representative ME-LWA produced using the resin-granite powder coating described in the previous section. The ME-LWAs exhibit an almost spherical shape and homogeneous outer shell, which is the result of the constant rotation of the samples in the granite powder during the coating process. The minimum and maximum diameter were determined to be 8.00 mm and 11.50 mm, respectively. The shell thickness was identified as 0.80 mm on average. Table 3 lists the main characteristics of the ME-LWA, while Figure 3 shows their internal pore size distribution before impregnation.

### 3.2. Mechanical Strength

**Figure 4:**
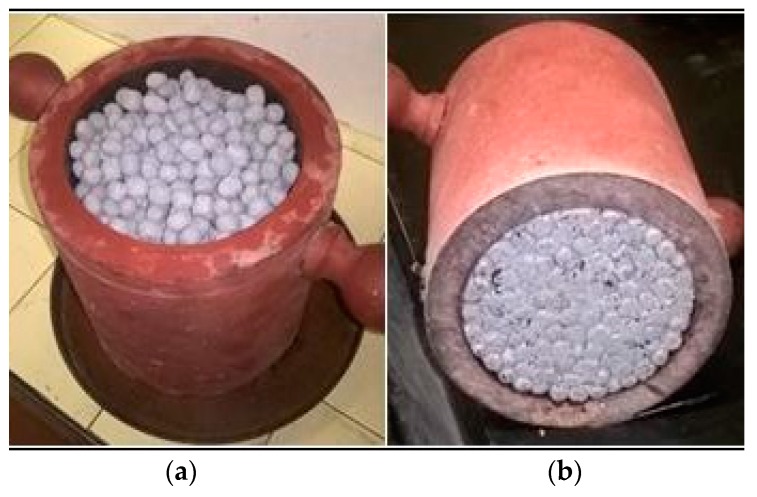
Bulk crushing resistance test of (**a**) before and (**b**) after testing.

The mechanical strength was evaluated by determining the bulk crushing resistance in accordance with EN 13055 [18]. The bulk crushing resistance can be carried out by means of two different procedures, depending on the dimension of the LWA and the weight of the sample. In the case of the manufactured ME-LWAs, Procedure 1 from the standard was chosen since the bulk density was above 150 kg/m^3^. A sample of the ME-LWAs was placed in a steel cylinder of one-litre volume (Figure 4); a piston was then pressed into the cylinder to a depth of 20 mm in a 100 s time interval; the force was recorded in Newtons. This procedure was repeated on three test specimens.

The crushing resistance, C_b_, of each sample was evaluated as follows:

C_b_ = (L + F)/A [N/mm^2^], (1)

where L is the force exerted by the piston [N], F is the compressive force [N], and A is the piston area [mm^2^].

Table 4 presents the calculated average crushing resistance of the ME-LWAs and raw LWAs. The ME-LWAs were determined to have an average crushing resistance of 9.24 MPa, which, compared to raw LWAs, was more than 7 times higher. This demonstrates that the polyester resin coating considerably increased the resistance of the final ME-LWA product. The Lightweight Expanded Clay Aggregates handbook shows several paving solutions incorporating different types of expanded clay aggregates. For porous asphalt pavements, for instance, a C_b_ value of 4.50 MPa is required, which is approximately half that recorded for the ME-LWAs.

### 3.3. ME-LWA Thermal Stability

**Figure 5:**
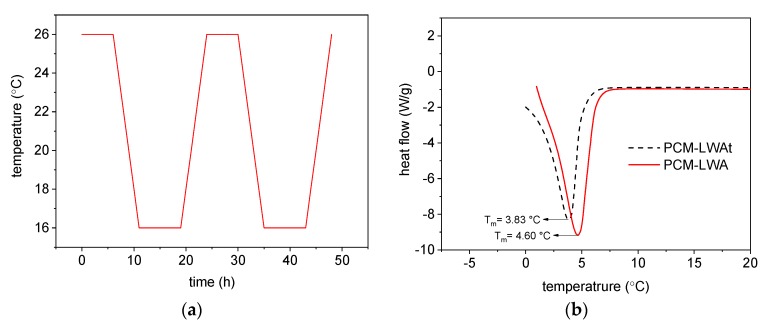
(**a**) Typical 48 h heating and cooling cycle and (**b**) DSC curves of ME-LWA before and after thermal cycling.

To ensure the reliable and consistent performance of the ME-LWA, the PCM encapsulated within the pores of the host material should be thermally reliable over many melting and freezing cycles and show little or no change in thermal properties after a long period of service. Therefore, the ME-LWA were subjected to melting/solidifying cycles in a temperature- and humidity-controlled environmental chamber to detect if there was any change in the phase change behaviour of the PCM and further verify the encapsulation efficiency. Figure 5a shows the typical two-day, time vs. temperature cycle, which lasted 5 weeks. The thermal properties, i.e., phase change temperature and latent heat of the PCM after repeated thermal cycling, were investigated by DSC. The DSC curve of the PCM before and after thermal cycling is shown in Figure 5b. When comparing the melting temperature of the PCM in the ME-LWA before and after thermal cycling, the melting temperature changes only by 0.7 °C while the latent heat storage capacity at melting changes by 12.56 kJ/kg. The change in mass of the 200 g sample of ME-LWA was also only 0.9 g. The changes observed in the thermal characteristics of the PCM contained in the LWA are very small; it can, therefore, be concluded that the prepared ME-LWA is thermally reliable.

### 3.4. Thermal Effectiveness of ME-LWA

**Figure 6:**
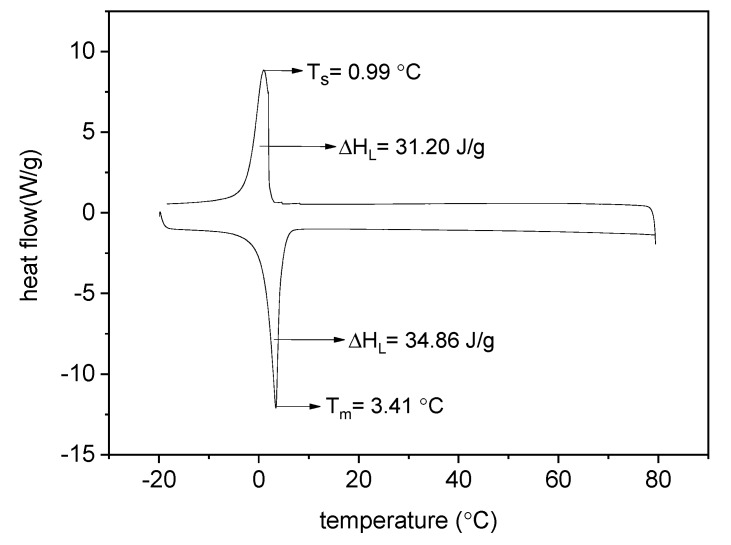
Thermal analysis test of the ME-LWA.

The differential scanning calorimetry (DSC) test was carried out with the aim of investigating the phase changing behaviour, particularly the phase change temperature and the thermal energy storage and release of ME-LWAs. The DSC curve of the ME-LWA in Figure 6 is constant between +20 °C and +80 °C, indicating that no significant variation of heat flow occurs and that the material remains stable without storing or releasing heat. However, in the range between 0 °C and 4 °C, the phase change rises, as expected, during both the melting and solidification cycles. The maximum heat flow recorded during the melting cycle occurred at 3.41 °C. Meanwhile, during the solidification cycle, the maximum heat flow occurred at 0.99 °C. The specific enthalpies of the phase changes were recorded as 34.86 J/g and 31.20 J/g for the melting solidification cycles, respectively.

**Figure 7:**
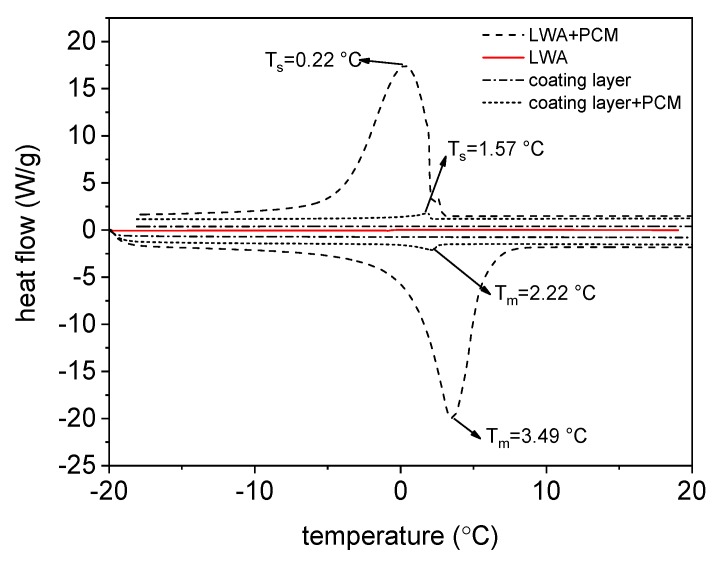
Thermal analysis tests of ME-LWA, the raw LWA and the resin coating.

Figure 7 shows the results of DSC tests of the raw and PCM-impregnated LWA from two different samples of the coating layer, from which the melting temperature (T_m_) and solidification temperature (T_s_) were calculated. T_m_ and T_s_ represent the melting and solidification values, respectively, at the peak points of the DSC curve, i.e., the values at which the highest heat flow is reached. The resin-granite powder coating layer proved to be completely insensitive to the temperature variation. The raw LWA, being composed of clay previously treated at 1200 °C, also showed the same neutral thermal behaviour. A slightly different result was obtained from the sample composed of the internal coating layer in contact with the paraffin: very narrow peaks appeared during both the heating and cooling process, showing a T_P_ = 2.22 °C and ΔH_m_ = 0.9225 J/g during the melting phase and T_P_ = 1.57 °C and ΔH_c_ = 1.045 J/g during the solidification phase. These outcomes attest to the remarkable thermal capacity of PCM: just a very small amount of this material induces an enthalpy alteration in the system. Finally, very broad and sharp peaks were displayed by the PCM-impregnated LWA: concerning the solidification phase change, T_S_ = 2.04 °C, T_P_ = 0.28 °C and ΔH_c_ = 62.44 J/g. Nonetheless, the high latent heat obtained is expected to decrease for the coated specimens.

**Figure 8:**
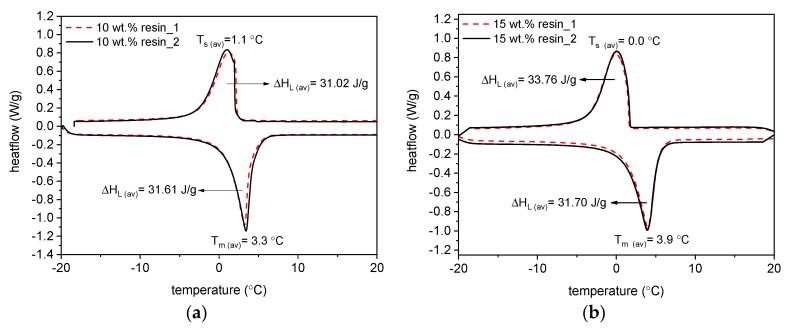
DSC tests of ME-LWAs samples from −20 °C to +20°C coated with (**a**) 10 wt % resin and (**b**) 15 wt % resin.

DSC tests on ME-LWA aggregates were carried out for two sets of samples coated using different quantities of resin. Figure 8a illustrates the results of two ME-LWA specimens coated with 10 wt % resin. Both specimens (taken from different ME-LWAs) revealed almost identical behaviour: the two curves are seen to be overlapped, with approximately the same amount of latent heat available at both melting and solidification, i.e., 31.61 J/g and 31.02 J/g, respectively. The mean peak melting and solidification temperatures were also almost identical, averaging at T_m (average)_ = 3.3 °C and T_s (average)_ = 1.1 °C, respectively. Figure 8b illustrates the results of two ME-LWA specimens coated with 15 wt % resin. Comparing the two sets of curves, melting of the PCM occurred at a higher temperature than for the 10 wt % resin coated ME-LWA samples, whereas solidification occurred at a lower temperature. In fact, the mean values of the melting and solidification temperatures were T_m (average)_ = 3.9 °C and T_s (average)_ = 0.0 °C, respectively. The latter offset in melting and solidification temperature is due to the increased resin content. Concerning the amount of latent heat, both specimens were characterized by sharp and broad bell-shaped curves, with the amount of latent heat available at melting and solidification equal to 31.70 J/g and 33.76 J/g, respectively.

The transition in the solid-liquid phase change between 1 °C and 3 °C asserted by the PCM manufacturer’s datasheet is in good agreement with the experimental results presented in Figure 8. The almost perfect overlap of the curves for both the 10 wt % and 15 wt % samples attests to the excellent reproducibility and repetitiveness of the recorded melting and solidification phases obtained using the DSC technique. These figures indicate that both sets of ME-LWAs exhibit similar thermal characteristics. This is because there is no chemical reaction between the paraffin PCM and the resin coating during the preparation of the ME-LWAs.

The specific theoretical enthalpy, H, can be calculated as H = ΔH × P [J/g], where ΔH is the specific phase change enthalpy of PCM, obtained from DSC test or directly from the datasheet, and P is the percentage in weight of paraffin hosted in LWA. The latent heat of PCM is provided by the supplier with an inaccuracy of 7.5%: its value was estimated at 185 J/g in the worst case. Considering that the PCM absorption capacity by vacuum impregnation was calculated as 95%, the theoretical enthalpy of the PCM hosted in a specific amount of LWA should be 175.75 J/g. Comparing this result with the value of specific enthalpy of impregnated aggregates without coating calculated by DSC test, equal to 62.44 J/g, it is clear that the heat transfer efficiency of ME-LWA was reduced. The lower thermal conductivity of the ME-LWA, along with the complex dispersion of the pores where the PCM is collected, undesirably affected the energy storage and release. This reduction is even more evident considering that the presence of the coating: the mean latent heat of the samples discussed above, is 32 J/g. The presence of polyester resin and granite powder contribute to the reduction of the ME-LWA specific enthalpy by approximately 50%.

### 3.5. ME-LWAs’ Resistance to Fragmentation

Many aggregates are more susceptible to abrasion when wet than dry [19]. The Micro-Deval test incorporates the use of water, in contrast to some other tests that are conducted on dry aggregates. The test results are helpful for evaluating the toughness/abrasion resistance of coarse aggregate subject to abrasion. In the EN 1097-2 standard of the Micro-Deval test [20], a Micro-Deval coefficient, which is the percentage of the original sample reduced to a size smaller than 1.6 mm during abrasion, is determined. However, in this study, the factor to be determined was the percentage of the original sample that did not incur any damage during the test. Hence, the procedure adopted here can be considered a variation of the EN 1097-1 standard. In terms of sample preparation, a sample of ME-LWA was weighed to the nearest gram and recorded as ‘A’. The sample was first soaked in pure water for 1 h, then transferred to the Micro-Deval container with the steel charges. The test was run for 120 min at 80 RPM, resulting in a total of 9600 revolutions. After the test was complete, the sample was separated from the steel balls and washed with water until the runoff was clear and all materials smaller than the 125-micron sieve had passed through. The steel charge was removed manually, and the ME-LWA was then air-dried. Damaged ME-LWAs were determined visually and subsequently removed, and the mass of the undamaged ME-LWAs was recorded as ‘B’. The percentage mass of ME-LWA that was left undamaged from the Micro-Deval abrasion test was calculated to the nearest 0.1%.

**Figure 9:**
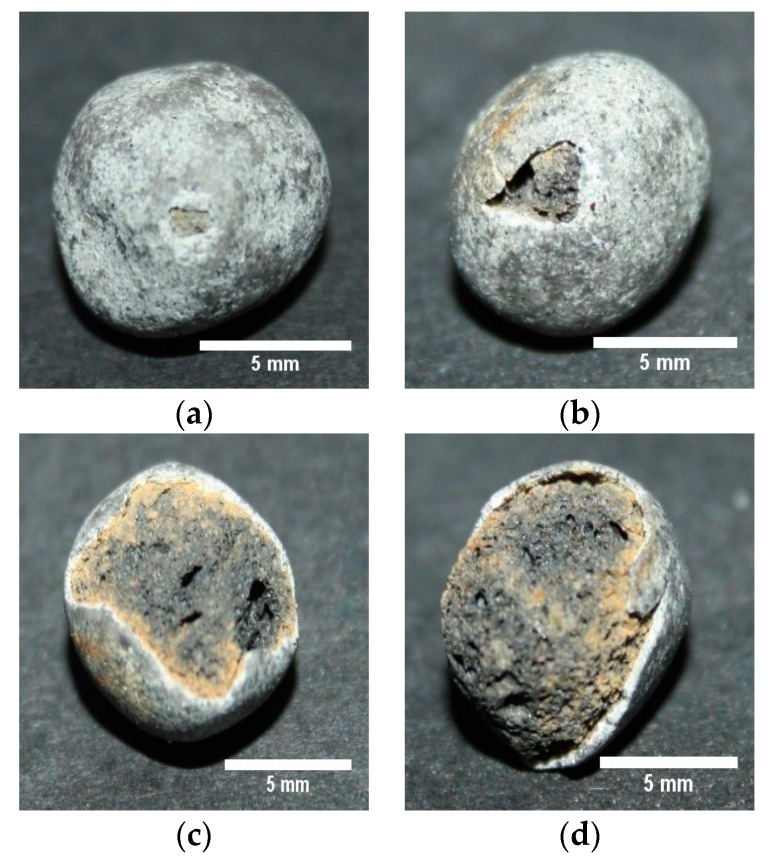
Fragmentation damage after Micro-Deval Showing (**a**) small pits (**b**) large pits (**c**) intermediate loss of coating and (**d**) significant loss of coating.

Some typical types of damage can be seen in Figure 9. The ME-LWAs suffered different degrees of damage, ranging from small and large pits in the coating (Figure 9a,b, respectively) to complete fracture with significant loss of coating (Figure 9c,d). 

The mass of sample that did not incur any damage was determined to be 600 g, hence A = 1000 g and B = 600 g, then % ME-LWA damaged = 40%. The interpretation of this result is straightforward and indicates that under the most severe conditions of wet abrasion, 40% of the ME-LWAs suffered some form of damage that could lead to the leaking of PCM. Under more realistic conditions, e.g., during the mixing of a cementitious binder, the ME-LWAs would not undergo such severe conditions.

### 3.6. Surface Polishing Characteristics of ME-LAWs (Micro-Roughness and Friction under Traffic)

**Figure 10:**
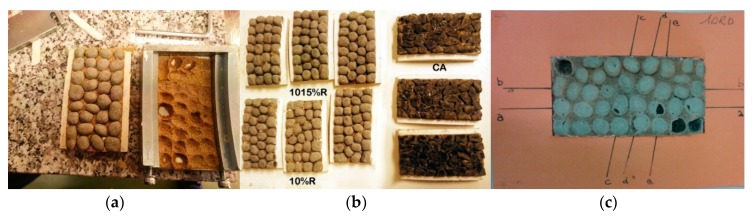
Specimens preparation for polishing test: (**a**) specimen demoulding; (**b**) sets of the experimentation specimens before polishing; (**c**) an example of superficial fragmentation of some granules after polishing test for a 10%R sample.

Surface micro-roughness parameters and the Polished Stone Value coefficient (PSV) were carried out on ME-LWAs specimens by means of a laser profilometer scanning [21] according to EN 1097-8 [22]. The polishing cycle involved two phases of 3 h duration. In the first phase, coarse abrasive sand (size 300/600 μm) and water were used; in the second phase, fine abrasive sand (smaller than 53 μm) was used instead, together with water. Two different types of aggregates were produced: ME-LWAs with 10% resin coating (ID sample: 10%R) and ME-LWAs with a first coating of 10% resin and a second coating of 15% resin (ID sample: 1015%R). Results were compared with a control non-coated aggregate (basalt, ID sample: CA) with high performance according to common road construction specifications [23,24]. Three specimens were prepared for each type of aggregate (10%R, 1015%R and CA), for a total of nine specimens (Figure 10a,b).

It is important to point out that a superficial fragmentation of some granules was observed for some of the 10%R samples (with a single resin coating) because they did not resist the smoothing action during the polishing test. This fragmentation was estimated to be around 16% of the surface area; however, this condition allowed the experimentation to be carried out correctly (Figure 10c).

#### 3.6.1. Roughness Analysis

**Figure 11:**
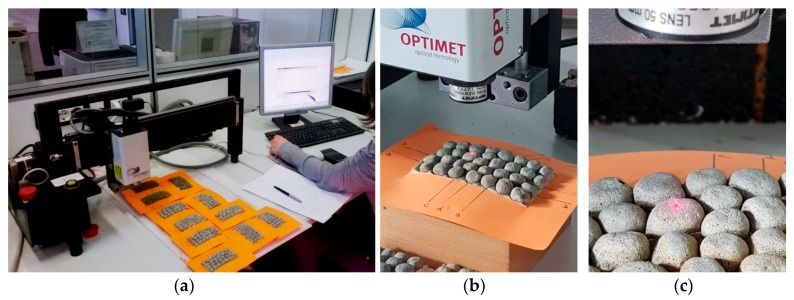
(**a**,**b**) Laser profilometer based on conoscopic holography used in the experimentation and (**c**) detail of the laser beam incidence on the surface of the granules.

**Figure 12:**
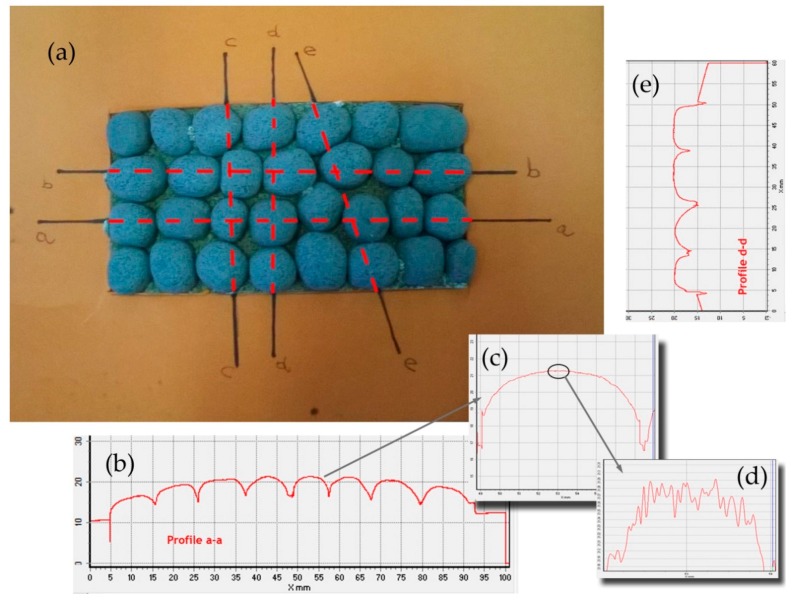
(**a**) Laser profilometer profile directions (**b**) profile along polishing direction a-a (**c**) zoom of granule surface (**d**) microprofile of granule surface (**e**) profile along crosswise direction d-d.

As far as roughness analysis is concerned, a laser profilometer based on conoscopic holography was used [21,25,26] (Figure 11). Conoscopic holography is a non-contact digitizing technique used in submicrometric roughness measurements. This is in contrast to laser triangulation techniques, which are the most common solution for this kind of measurement, but there are fundamental limitations to their applicability when high precision, long standoffs or large apertures are needed, such as in this study [27]. For each aggregate sample, five profiles (a, b, c, d and e) were analysed on the top layer of the polished stone specimens. Two profiles along the same direction of polishing and three in the crosswise direction were carried out before and after the polishing test (Figure 12a). Micro-roughness parameters (Ra, Rq and Rz) [28] were derived from the profile analysis, as reported in Table 5. In the literature it is also possible to calculate these parameters through laser measurements [29]. These technologies are also applicable on a different scale for measurements related to deterioration, road geometry and contact tire-surface [30,31,32].

Table 6 summarizes the roughness parameters before and after the polishing test. These values were derived from microprofiles sampled on the top of each aggregate with an average length (base-line) of 0.95–1.50 mm (Figure 12d). Furthermore, the percentage decreases between the two monitoring steps (before-after) were estimated.

According to Table 6, for all samples (CA, 10%R and 1015%R), the depth parameters decrease after polishing, with the lowest percentage decrease (on average −5%) being recorded for sample CA. Also, samples with a double layer of resin (1015%R) are more resistant to polishing (average decrease −14%) compared to 10%R (on average −20%).

#### 3.6.2. Polished Stone Analysis

**Figure 13:**
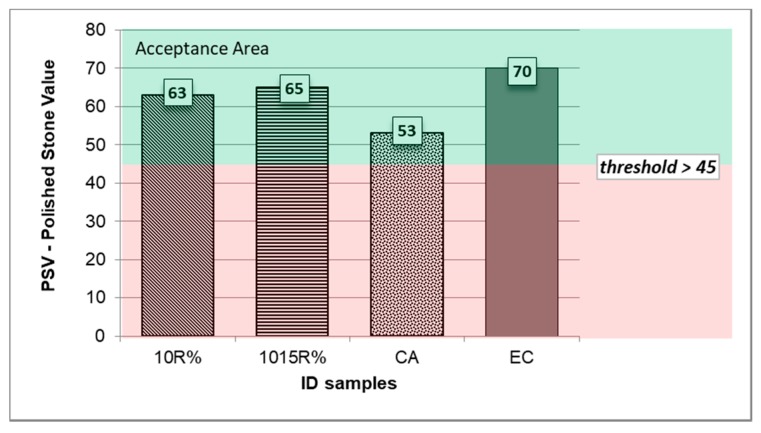
Histogram of average Polished Stone Values for samples 10%R, 1015%R and CA compared with PSVs of expanded clay and acceptance threshold values (green area) from common Italian technical specifications. (Note: in the figure PSVs are × 100.).

The residual friction after the polishing test was also investigated in terms of PSV, for which the outcomes are shown in Table 7 and Figure 13. In order to better investigate the PSV variability, the performance of expanded clay (ID sample: EC) was considered. Usually, EC is used for the surface layer of anti-skid and anti-noise asphalt pavements. Table 7 shows the acceptance threshold values for the polishing test according to widely adopted Italian technical specifications, too.

As shown in Table 7 and Figure 13, the average values of the PSV coefficient (multiplied by 100) for both types of ME-LWAs are higher than those registered for the control aggregate. More specifically, PSV is recorded to be 53 for CA versus 63 for 10%R samples and 65 for the 1015%R sample. Finally, the average PSV registered for ME-LWAs is always higher than the specifications’ acceptance thresholds. Based on the abovementioned findings, it is possible to highlight that the double surface-coating technique (1015%R sample) allows for the attainment of a better performance than the aggregates made with a single-coating for road pavements in terms of both micro-roughness and friction development under traffic loadings.

### 3.7. Thermal Analysis of Relevant Temperature Conditions

Three further thermal measurement tests were carried out to obtain a more realistic evaluation of the ME-LWA effectiveness for their use as anti-icing solution in pavements. Two different samples of ME-LWAs and one sample of the raw LWA were prepared and tested in both a climatic chamber and a cold outdoor environment, monitoring their thermal behaviour as a result of the controlled and uncontrolled temperature reductions, respectively. Temperature measurements in the climatic chamber were performed using a FLIR thermal camera, infrared thermometer and thermocouples, while ambient temperature monitoring was conducted using a digital thermometer.

**Figure 14:**
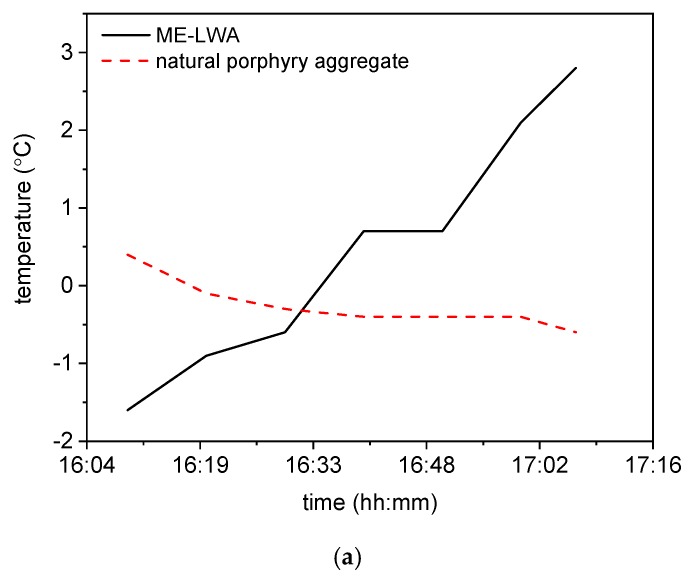

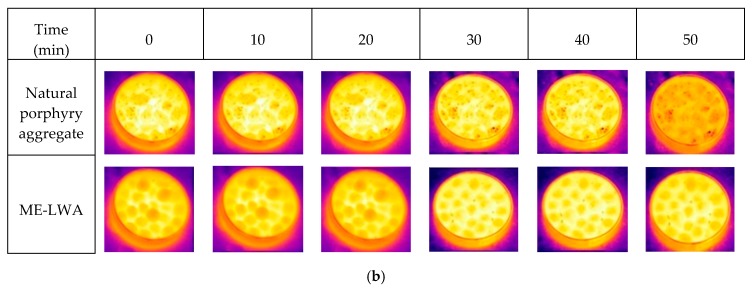
(**a**) Temperature differences versus time and (**b**) thermal images of natural porphyry aggregate and ME-LWAs over a period of 50 minutes.

The measurements in both environments were performed for a sufficient time, during which sub-zero temperatures were reached. The FLIR thermal camera provided thermal pictures of the samples, from which the development of the temperature as a function of time was plotted (Figure 14). The two samples made of ME-LWA are placed on the left and right of the thermal pictures, respectively, while the natural porphyry aggregate is placed in the centre. Pictures are taken at 10-min intervals from 0 to 50 min.

During the first 30 min, the differences between the ME-LWA samples and the natural aggregate were negative, meaning that the ME-LWAs were colder. However, with the decrease in the temperature, between 30 min and the end of the test, the values became positive, showing that the ME-LWAs maintained higher temperatures than the natural aggregate, reaching a maximum value of 2.8 °C. This trend was also observed from the thermal images presented in Figure 14b, which clearly show the remarkable effect of PCM heat flow, particularly in the last 30 min, where the ME-LWAs can be seen to be glowing much brighter than the natural porphyry aggregates.

**Figure 15:**
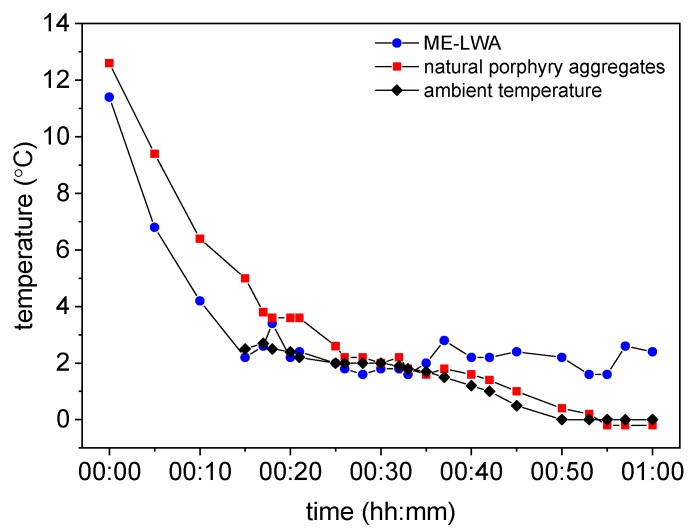
Development of temperature as a function of time using the infrared thermometer.

The measurement with the infrared thermometer (Figure 15) also shows a different evolution of the temperature: after 25−30 min, the ME-LWAs remained approximately constant and close to 2 °C, while the natural porphyry aggregates continued to decrease, reaching a temperature of 0 °C.

**Figure 16:**
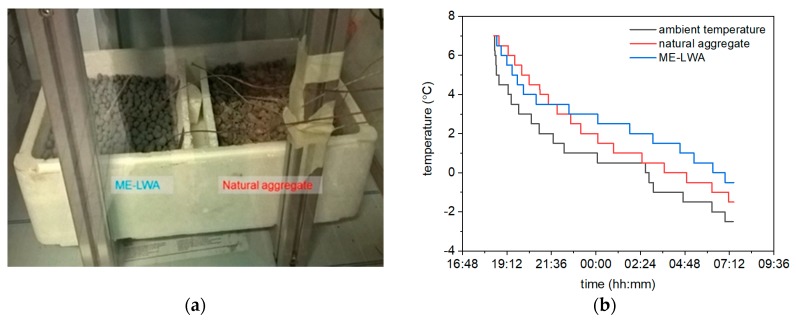
(**a**) Climatic chamber recording the temperature of ME-LWA and porphyry aggregates and (**b**) development of temperature versus time inside the climatic chamber.

An additional test was carried out in a climatic chamber to evaluate how long ME-LWAs were able to maintain temperatures above 0 °C. For this experiment, neither the thermal camera nor the infrared thermometer was used since the glass surfaces of the chamber would influence the measurements. Instead, digital thermocouples were embedded into samples of ME-LWAs and natural porphyry aggregates (Figure 16). This analysis was performed to simulate a typical local temperature drop during the night; the chamber temperature was manually controlled to reproduce a set of available field data from the nearby climate station.

Figure 16 shows the fitted time versus temperature curves of the chamber air, natural aggregates and ME-LWA. The curve for the ME-LWA shows attenuation in its slope starting from 3−4 °C, while the natural aggregates continue to decrease in temperature, following almost the same trend as the chamber temperature. The meaningful difference in their behaviour was shown between approximately 04:30 and 07:00, when the sample temperature was below 0 °C. The ME-LWA, on the other hand, maintained a temperature above 0 °C, more likely due to the PCM phase change from liquid to solid and subsequent exothermic heat flow. 

The latter results are promising since this material has been demonstrated to have a remarkable delaying effect on the temperature drop as soon as 3 °C is reached. It is acceptable to assume that the presence of bitumen and other virgin aggregates in an asphalt mix would hinder PCM heat release; however, it is still likely that ME-LWA aggregates would slow down or prevent the formation of ice on road surfaces where the climate is not that harsh.

## 4. Conclusions

This preliminary work aims at the assessment of macro-encapsulated lightweight aggregates containing PCM for paving application purposes. In light of the above, the following concluding remarks can be made:

- The production of ME-LWA was proven feasible at a laboratory scale and could be implemented on an industrial level by taking into account the described variables;
- The geometrical and mechanical properties of the LWA are not affected by the macro-encapsulation process. The resin coating provides additional strength to the aggregates;
- The PCM is thermally stable, showing only a 0.7 °C change in melting temperature after thermal cyclic testing. The resin coating undoubtedly reduces the thermal effects of the pure PCM. Nonetheless, an average latent heat of 32 J/g can still be achieved.
- The strength of coating is a key point for the durability of the aggregates with traffic: a double coating of resin was proven effective against polishing, producing an aggregate with only a 9% lower polishing resistance than the reference basalt aggregate.
- The thermal effects on aggregate temperature were tested in a relevant climate using different sensors and temperature gradients with time: the ME-LWAs temperature was measured and was proven to be effective at maintaining a temperature above 0 °C.

## Figures and Tables

**Table 1:** Thermophysical properties of technical grade paraffin PCM (obtained from manufacturer).

Properties	Values
Phase change range temperature [°C]	1–3
Thermal energy storage capacity [kJ/kg]	200 (±7.5%)
Specific heat capacity [kJ/kg∙K]	2
Liquid density at 15 °C [kg/m^3^]	0.77
Solid density at −15 °C [kg/m^3^]	0.88
Thermal conductivity [W/m∙K]	0.2
Maximum operation temperature [°C]	60

**Table 2:** Physical properties of Palatal P4-01 polyester resin.

Properties	Values
Viscosity at 23 °C [MPa∙s]	540–610
Density at 20 °C [kg/m^3^]	1100
Cure time from 25 °C to 35 °C [min]	16–21
Maximum operation temperature [°C]	135–155

**Table 3:** Properties of the impregnated and coated LWA.

Properties	Values
Loose bulk density [kg/m^3^]	274 (±15%)
Thermal conductivity [W/m∙°C]	0.10
Water absorption capacity [wt %]	0.055
PCM absorption capacity by vacuum impregnation (1 h) [wt %]	95
Average diameter [mm]	8.00
Average shell thickness [mm]	0.80

**Table 4:** ME-LWA crushing resistance.

Parameter	Sample 1	Sample 2	Average	Raw LWA
Piston weight [kN]	20.48	20.48	20.48	
Piston area [mm^2^]	11,309.73	11,309.73	11,309.73	
Maximum load [daN]	10,875	10,010	10,442	
C_b_ [N/mm^2^]	9.62	8.85	9.24	1.30

**Table 5:** Roughness analysis by laser profiles post-processing: descriptions and formulas of roughness parameters derived from microprofiles.

Roughness Parameters	Description	Formula	
Ra	Arithmetical mean deviation of the assessed profile	$R_{a}=\frac{1}{n}\overset{n}{\underset{i=1}{\sum }}\left|y_{i}\right|$	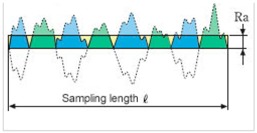
Rq	Root mean squared	$R_{q}=\sqrt{\frac{1}{n}\overset{n}{\underset{i=1}{\sum }}y_{i}{}^{2}}$	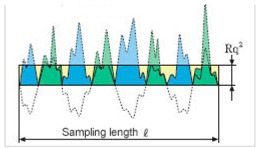
Rz	Parameter based on the five highest peaks (R_pi_) and lowest valleys (R_vi_) over the entire sampling length	$R_{z}=\frac{1}{5}\overset{5}{\underset{i=1}{\sum }}R_{pi}-R_{vi}$	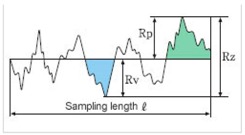

**Table 6:** Roughness analysis by laser profiles post-processing: roughness parameter values before and after polishing test.

**ID Sample: CA**	**Longitudinal and Cross Profiles**								
	**Before**			**After**			**Depth Loss (−) or Depth Increase (+)**		
Profile roughness parameters	Ra	Rq	Rz	Ra	Rq	Rz	Ra	Rq	Rz
Average [μm]	15.3	19.7	42.8	14.5	18.7	41.5	**−6%**	**−5%**	**−3%**
Number of microprofiles analysed	180								
Length baseline of profile [μm]	Average = 1130; Max = 2800; Min = 300								
**ID Sample: 10R%**	**Longitudinal and Cross Profiles**								
	**Before**			**After**			**Depth Loss (−) or Depth Increase (+)**		
Profile roughness parameters	Ra	Rq	Rz	Ra	Rq	Rz	Ra	Rq	Rz
Average [μm]	13.8	17.5	40.9	11.1	13.9	32.0	**−20%**	**−20%**	**−22%**
Number of microprofiles analysed	167								
Length baseline of profile [μm]	Average = 1506; Max = 2250; Min = 490								
**ID Sample: 1015R%**	**Longitudinal and Cross Profiles**								
	**Before**			**After**			**Depth Loss (−) or Depth Increase (+)**		
Profile roughness parameters	Ra	Rq	Rz	Ra	Rq	Rz	Ra	Rq	Rz
Average [μm]	11.9	15.3	34.4	10.4	13.2	28.7	**−12%**	**−13%**	**−17%**
Number of microprofiles analysed	188								
Length baseline of profile [μm]	Average = 948; Max = 1440; Min = 490								
Delta X [μm]	10								

**Table 7:** Average Polished Stone Values for samples tested (10%R; 1015%R and CA) compared with PSVs of expanded clay and acceptance threshold values from Italian specifications (PSVs x 100).

ID	10%R	1015%R	CA	EC	Specification
**PSV**	63	65	53	70	≥45−50
